# Neurophysiological Aspects in SARS-CoV-2–Induced Acute Respiratory Distress Syndrome

**DOI:** 10.3389/fneur.2022.868538

**Published:** 2022-05-16

**Authors:** Eleonora Vecchio, Lara Gallicchio, Nicola Caporusso, Valentina Recchia, Luigi Didonna, Giancarlo Pezzuto, Luigi Pisani, Antonella Petruzzellis, Vito Delmonte, Filippo Tamma

**Affiliations:** ^1^Department of Neurology, General Regional Hospital “F. Miulli”, Acquaviva delle Fonti, Bari, Italy; ^2^Department of Intensive Care, General Regional Hospital “F. Miulli”, Acquaviva delle Fonti, Bari, Italy

**Keywords:** COVID-19, ARDS, brainstem, nervous system involvement, Guillain-Barré syndrome

## Abstract

Patients with coronavirus disease 2019 (COVID-19) often develop acute respiratory failure and acute respiratory distress syndrome (ARDS) that requires intensive care unit (ICU) hospitalization and invasive mechanical ventilation, associated with a high mortality rate. In addition, many patients fail early weaning attempts, further increasing ICU length of stay and mortality. COVID-19 related ARDS can be complicated by neurological involvement with mechanisms of direct central nervous system (CNS) infection and with overlapping para-infective mechanisms of the peripheral nervous system (PNS). We aimed to evaluate the possible involvement of the brainstem and PNS in patients with COVID-19 related ARDS and difficulty in weaning from mechanical ventilation. We evaluated electroencephalogram (EEG), brainstem auditory evoked potentials (BAEPs), electroneurography of the four limbs and the phrenic nerve in 10 patients with respiratory insufficiency due to SARS-CoV-2. All were admitted to intensive care unit and were facing prolonged weaning from mechanical ventilation. All ten patients showed a mild diffuse non-specific slowing of brain electrical activity on the EEG. Four patients had an acute motor axonal neuropathy with absent or reduced amplitude phrenic nerve CMAP while four patients showed impairment of the BAEPs. A patient with peripheral nerve impairment suggestive of Guillain-Barré syndrome (GBS) underwent an intravenous immunoglobulin (IVIg) cycle that led to an improvement in the weaning process and progressive motor improvement. The inclusion of a comprehensive neurological evaluation in COVID-19 patients in ICU facilitated the early identification and effective management of Nervous System involvement.

## Introduction

Interstitial pneumonia due to SARS-CoV-2 can be complicated by possible neurological involvement with mechanisms of direct CNS infection and/or with para-infective mechanisms of the peripheral nervous system (PNS), shown by some neuropathological findings of COVID-19 patients, as recently reviewed (1).

Respiratory failure appears to be one of the most worrying complications due to SARS-CoV-2 infection. Patients can develop severe pneumonia that requires invasive mechanical ventilation that leads to death in a significant percentage of them. Furthermore, many patients fail early weaning attempts, thus prolonging the length of stay in the intensive care unit (ICU) and increasing in that way complications, morbidity, and mortality. In some cases, there appears to be a discrepancy between the severity of lung involvement and respiratory function. Severe COVID-19 leads to death through multiple mechanisms, including myocardial damage, renal failure, shock, and disseminated intravascular coagulopathy (2, 3). It has also been suggested that the brain stem could play a role in the severe respiratory failure of COVID-19 patients (4). This hypothesis comes from animal models infected with other coronaviruses that have shown that the brainstem is severely affected and in particular the respiratory center (i.e., the nucleus of the solitary tract in the medulla oblongata) (5). In a small case series of an Italian group, this hypothesis is taken into consideration in patients with poor recovery of respiratory function when SARS-CoV-2 pneumonia improves (6). The EEG of these patients showed a diffuse slowing while the brain CT or MRI evaluation was substantially normal (6). In a neurophysiological evaluation using Blink-Reflex in 11 patients with typical interstitial pneumonia due to COVID-19 and severe respiratory failure, the authors highlighted the absence or alteration of the RII component, suggesting a possible involvement of the brainstem, especially at the level of the bulb (7).

Recent reports describe a Guillain-Barré syndrome related to SARS-CoV-2, characterized mainly by axonal impairment with early involvement of cranial nerves that could lead to severe respiratory failure (8).

Our aim was to consider the possible overlap of central and peripheral nervous system involvement in patients with respiratory insufficiency due to COVID-19 and difficulty in weaning from mechanical ventilation.

## Materials and Methods

We present data about patients with respiratory failure due to SARS-CoV-2 infection admitted to the intensive care unit of the *Miulli Hospital in Acquaviva delle Fonti* in the period from 1 March to 30 May 2021, evaluated because they were facing prolonged weaning from mechanical ventilation, despite the improvement in pulmonary conditions. All patients underwent electroencephalogram (EEG), brainstem auditory evoked potentials (BAEPs), electroneurography of the four limbs and the phrenic nerve. A standard 20 min EEG was recorded according to the 10–20 International system of electrode placement. BAEPs were recorded following auditory stimulation by a 100-μs 85-dB ± click applied to one ear, with a (−20 dB) contralateral masking using “white noise.” The recurrence frequency was 11 Hz (bandpass, 150–1,500 Hz; sweep time, 10 ms). Two sets of 2,000 sweeps were averaged. BAEPs were picked up in Cz. The reference electrode was placed at the earlobe ipsilateral to the stimulated ear. Nerve conduction studies were performed according to standardized techniques. Distal motor latency, amplitude and duration of negative peak of compound muscle action potential (CMAP), motor conduction velocity and minimal F-wave latency were measured from different stimulation sites (median, ulnar, peroneal, tibial, and phrenic nerves). Sensory studies were performed anti-dramatically in median, ulnar and sural nerves and amplitude of sensory nerve action potential was measured baseline to negative peak. Patients with previous pathology of the central and peripheral nervous system were not considered. At the time of the evaluation, patients were subjected to only mild sedation with dexmedetomidine.

## Results

**Figure 1:**
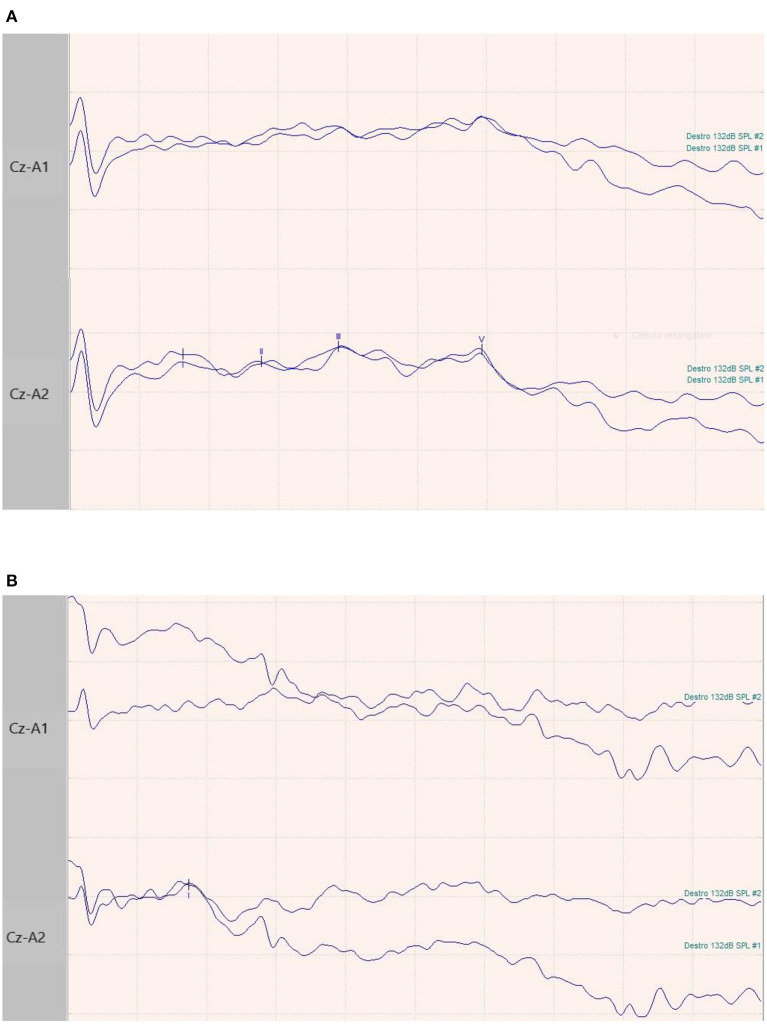
BAEPs recordings in two patients. **(A)** 65-year-old male patient with normal I-III-V complex latency and amplitude and related inter-peak times. **(B)** 73-year-old female patient with normal latency and amplitude of I complex, and absence of III and V waves.

We evaluated 10 patients, 5 male, aged 53–75 years (mean 66.1); the duration of Covid, from the first detection of SARS-CoV-2 RNA in respiratory specimen (Swab Nasopharyngeal) until the neurophysiological evaluation, was 6–50 days (mean 25.5). In all subjects, the onset of symptoms was on the same day or the day before arrival in ED and the diagnosis of SARS-CoV-2 infection.The main comorbidities presented by the patients were hypertension (present in the 80% of subjects), obesity (60%), Chronic kidney Disease (20%), diabetes (20%). All ten patients showed a mild diffuse non-specific slowing of brain electrical activity on the EEG. Three patients showed normal BAEPs, while in two patients we found non-evocable responses and in 5 patients increased interpeak III-V wave latency monolaterally (Table 1, Figure 1). We performed extensive electrophysiological examination in all patients. Six of the patients examined show substantially normal or not significant findings.

**Table 1:** Brainstem evoked potential results obtained in the 10 patients evaluated (normal values referred to normative values of our laboratory).

	**Lat. I (R/L) (ms)**	**Lat. III (R/L) (ms)**	**Lat. V (R/L) (ms)**	**I-III (R/L) (ms)**	**III-V (R/L) (ms)**	**I-V (R/L) (ms)**
Normal values	1.7 ± 0.15	4.5 ± 0.2	5.7 ± 0.25	2.1± 0.15	1.9 ± 0.18	4 ± 3SD
Pat. 1	1.61/1.81	3.85/3.89	5.92/6.23	2.24/2.08	2.07/2.34	4.31/4.42
Pat.2	1.88/1.86	4/3.95	6.14/6.12	2.12/2.09	2.14/2.17	4.26/4.26
Pat. 3	1.73/1.79	3.93/3.67	5.79/ab	2.2/1.88	1.86/ab	4.06/ab
Pat. 4	ab/1.79	ab/3.26	ab/5.08	ab/1.47	ab/1.82	ab/3.29
Pat. 5	1.94/1.73	4.0/4.0	6.85/6.05	2.27/2.06	2.85/2.05	4.91/4.32
Pat 6	2.05/1.83	3.29/3.99	6.42/5.94	1.24/2.16	3.13/1.95	4.37/4.11
Pat 7	1.98/ab	4.46/ab	6.9/ab	2.48/ab	2.44/ab	4.92/ab
Pat. 8	1.74/1.65	3.8/3.25	5.76/5.39	2.06/1.6	1.96/2.14	4.02/3.74
Pat. 9	1.75/1.6	4.34/4.3	6.3/6.1	2.59/2.7	1.96/1.8	4.55/4.5
Pat. 10	1.7/1.5	3.9/3.9	5.9/5.8	2.2/2.4	2/1.9	4.2/4.3

**Figure 2:**
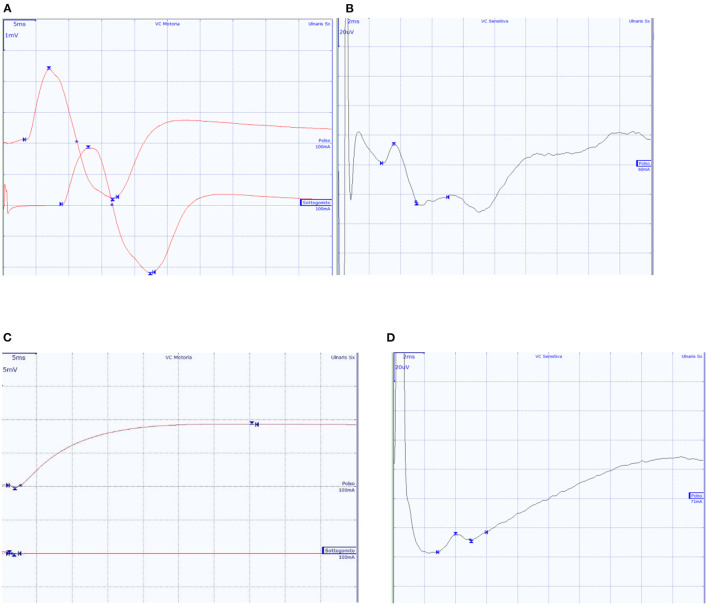
Ulnar nerve CMAP and SAP of two subjects. **(A,B)** A 65-year-old male patient with normal findings; **(C,D)** A 57-year-old patient with diffuse axonal neuropathy, with absent ulnar nerve CMAP, and SAP within normal values.

In 6 patients the neurological evaluation showed no specific or significant findings. In these patients the electroneurographic study showed normal findings or abnormal studies that did not allow a specific electrodiagnostic classification (Figure 2). Four of the 10 subjects showed rapidly progressive tetraparesis with areflexia. In these, the nerve conduction studies showed low or absent motor responses with preserved sensory responses (Supplementary Table 2, Figure 2), whilst needle EMG findings were consistent with intense and diffuse denervation. In these patients also the CMAP of the phrenic nerve was bilaterally not evocable. These findings suggested a clinical and neurophysiological diagnosis of acute motor axonal neuropathy (AMAN) (Supplementary Table 2), according to the Rajabally criteria (9). We managed to treat one of these four patients with an IVIg cycle (2 g/kg) which led to an improvement in the weaning process and progressive motor improvement. This patient was then discharged at home after 105 days because of renal complications. In the remaining three patients with GBS profile, two of them died, respectively, after 29 and 75 days; the third patient was transferred to the COVID-19 Respiratory sub-intensive unit, and, after 38 days of hospitalization, was sent to a rehabilitation center. The patients with non-GBS profile were sent to rehabilitation centers after a mean of 46 days of hospitalization.

## Discussion

Whilst respiratory failure in COVID-19 arises from severe interstitial lung involvement (10), SARS-CoV-2 likely spreads also through the nervous system. It might spread cell-to-cell in a prion-like way (5, 11, 12) along the vagus nerve, reaching respiratory centers in the brainstem, possibly adding a neurogenic component to the respiratory failure (4, 5). Seven of our studied patients presented alterations of the BAEPs, but in 3 of them due to non-evocable responses, it was not possible to exclude a preexisting hearing loss, suggesting a previous peripheral acoustic nerve disorder or technical pitfalls. The remaining four subjects showed a prolonged III–V inter-peak latency, suggesting changes between caudal pons and midbrain. These neurophysiological findings may be suggestive of SARS-CoV-2-related brainstem involvement in severe COVID-19 patients. EEG findings were non-specific and possibly related to the hypoxic and metabolic conditions of the patients, in addition to a possible pharmacological effect induced by dexmedetomidine.

Another important aspect that emerged from our evaluation in patients with COVID-19 is the need to consider a Guillain-Barré syndrome associated with severe respiratory impairment. Typically, GBS is a post-infectious condition with symptom onset for 76% of the patients occurring in about 4 weeks after the preceding respiratory or gastrointestinal infection (8). The para-infectious profile like the one described in our series, is an atypical feature that was only recently reported among patients infected with the Zika virus and SARS-CoV-2 (8). In other, larger series of patients with GBS associated with COVID-19, emerged a higher frequency of subjects in which GBS started while COVID-19 symptoms were still ongoing (13–15). Furthermore, COVID-GBS patients had respiratory symptoms at presentation to the ED, and the length of these symptoms was significantly longer than in COVID–non-GBS patient (14). The diagnosis of SARS-CoV-2 infection, the absence of any other immunological or microbiological explanation, and the epidemiological finding of increased relative frequency and standardized incidence of GBS in the COVID-19 patients in some studies, suggest that SARS-CoV-2 may have been responsible for the development of GBS in these patients (14, 15). The detection of a relatively high incidence of GBS cases, and in particular of AMAN subtype in our series, may therefore derive from the selection of patient only with a more severe COVID-19. This association, in subjects not specifically treated, revealed a worse outcome in our series. Accurate identification and categorization of GBS patients are very important, since the para-infectious profile is associated with a concurrent manifestation of COVID-19 and GBS symptoms, which can complicate the treatment and may be associated with a worse prognosis. Indeed, the patients with a para-infectious profile were more likely to have a poor prognosis (8, 13).

This study has several limitations. First, the number of cases was very small. Second, it is a retrospective study and some findings such as antiganglioside antibody titres and cerebrospinal fluid analysis were not available. However, it should be considered that these patients were studied in a pandemic context and under the pressure of and exceptional health emergency in a hospital of southern Italy. Therefore, the interpretation of our findings should be made with caution and should be interpreted as hypothesis-generating.

## Conclusion

Overall, our results suggest that a central, mainly at brainstem level, and peripheral nervous system involvement likely contributes to respiratory failure in COVID-19 patients. The inclusion of a comprehensive neurological evaluation in SARS-CoV-2 patients with clinical and radiological lung amelioration, but difficulty in weaning from mechanical ventilation, facilitated the identification and effective treatment of neurological involvement.

## Data Availability Statement

The original contributions presented in the study are included in the article/Supplementary Material, further inquiries can be directed to the corresponding author.

## Ethics Statement

Ethical review and approval was not required for the study on human participants in accordance with the local legislation and institutional requirements. Patient's next of kin provided their written informed consent to participate in this study.

## Author Contributions

EV, LG, and NC: conception, design, and drafting article. VR, LD, GP, and LP: acquisition of data. EV and AP: analysis and interpretation of data. FT and VD: study supervision. All authors critically revised the article and reviewed final version of the manuscript and approved it for submission.

## Conflict of Interest

The authors declare that the research was conducted in the absence of any commercial or financial relationships that could be construed as a potential conflict of interest.

## Publisher's Note

All claims expressed in this article are solely those of the authors and do not necessarily represent those of their affiliated organizations, or those of the publisher, the editors and the reviewers. Any product that may be evaluated in this article, or claim that may be made by its manufacturer, is not guaranteed or endorsed by the publisher.
