# Supplementary material for: Neurophysiological Aspects in SARS-CoV-2–Induced Acute Respiratory Distress Syndrome
Source: Front Neurol. 2022 May 16;13:868538. doi: 10.3389/fneur.2022.868538 (PMC9149271; doi:10.3389/fneur.2022.868538)
Supplement: Supplementary file 1 [file Table_1.DOCX]

| patient 1 |  |  |  |  |  |  |  |  |  |  |  |  |  |
| --- | --- | --- | --- | --- | --- | --- | --- | --- | --- | --- | --- | --- | --- |
|  | nerve | MNCV (R/L) (m/s) | Normal MNCV | CMAP Amplitude (R/L) mV | normal Ampl | Motor latency (R/L) m/s | normal latency (R/L) m/s | SNCV (R/L) (m/s) | normal SNCV | SAP (R/L) | normal ampl | Sensitive Latency | normal sensitive Latency |
|  | median | ab/ab | ≥47 | ab/ab | ≥ 4 | ab/ab | ≤4,2 | 39,9/33,9 | ≥44 | 10,3/5,3 | ≥ 8 |  | ≤ 3,5 |
|  | ulnar | ab/ab | ≥47 | ab/ab | ≥ 4 | ab/ab | ≤ 3,6 | 44,2/44,6 | ≥44 | 7,9/3,5 | ≥ 8 |  | ≤ 3,5 |
|  | sural |  |  |  |  |  |  | ab/56,6 | ≥ 39 | ne/7 | ≥ 4 | ne/2,5 | ≤ 3,5 |
|  | phrenic | ab/ab |  | ab/ab |  | ab/ab | 7 ± 0,7 |  |  |  |  |  |  |
|  | tibial | ab/ab |  | ab/ab | ≥ 2,5 | ab/ab | ≤ 6,5 |  |  |  |  |  |  |
|  | peroneal | ab/ab |  | ab/ab | ≥ 2,5 | ab/ab | ≤ 5,5 |  |  |  |  |  |  |
|  |  |  |  |  |  |  |  |  |  |  |  |  |  |
| patient 6 |  |  |  |  |  |  |  |  |  |  |  |  |  |
|  | median | 49,1/52,4 | ≥47 | 1,9/0,4 | ≥ 4 | 4,2/3 |  | 48,1/55,2 | ≥44 | 13,6/18,8 | ≥ 8 | 3,3/2,5 |  |
|  | ulnar | ne/50,1 | ≥47 | ne/1,4 |  | 1,2/2,4 |  | ne/62 | ≥44 | ne/7,5 | ≥ 8 | 2,7/2,3 |  |
|  | phrenic |  |  | ab/ab |  | ab/ab |  |  |  |  |  |  |  |
|  | sural |  |  |  |  |  |  | ab/ab | ≥ 39 | ab/ab | ≥ 4 |  |  |
|  | peroneal | ne/52,8 | ≥ 39 | ne/3,2 | ≥ 2,5 | ne/1,2 |  |  |  |  |  |  |  |
|  | tibial | ne/49,3 | ≥ 37 | ne/1,3 | ≥ 2,5 | ne/1,2 |  |  |  |  |  |  |  |
|  | F-Wave |  |  |  |  | ab/ab |  |  |  |  |  |  |  |
| patient 7 |  |  |  |  |  |  |  |  |  |  |  |  |  |
|  | median | 51,4/ne | ≥47 | 0,8/ne | ≥ 4 | 4,2/ne |  | 57,2/47,6 | ≥ 44 | 5,93/15,3 | ≥ 8 | 1,9/2,6 |  |
|  | ulnar | 45,3/ne | ≥47 | 0,4/ne | ≥ 4 | 2,8/ne |  | 58,5/45,9 | ≥ 44 | 19,9/13,9 | ≥ 8 | 2,1/2,3 |  |
|  | phrenic |  |  | ab/ab |  | ab/ab |  |  |  |  |  |  |  |
|  | sural |  |  |  |  |  |  | 55,2/ab | ≥ 39 | 51,1/ab | ≥ 4 | 2,5/nv |  |
|  | tibial | 42,4/ab | ≥ 37 | 1,5/ab | ≥ 2,5 | 7/ab |  |  |  |  |  |  |  |
|  | peroneal | ab/ab | ≥ 39 | ne/ne | ≥ 2,5 |  |  |  |  |  |  |  |  |
| patient 8 |  |  |  |  |  |  |  |  |  |  |  |  |  |
|  | median | 35/44,3 | ≥47 | 0.4/3,4 | ≥ 4 | 5,9/5,1 |  | nv/36 | ≥ 44 | nv/5,8 | ≥ 8 | nv/5,9 |  |
|  | ulnar | 45/44,6 | ≥47 | 2,8/5,1 | ≥ 4 | 5,4/2,4 |  | 34/44 | ≥ 44 | 5,3/5,1 | ≥ 8 | 2,8/2,2 |  |
|  | sural |  |  |  |  |  |  | 59.5/53,3 | ≥ 39 | 15.9/14.8 | ≥ 4 | 2,4/2,5 |  |
|  | tibial | 28,6/ne | ≥ 37 | 1,2/ne | ≥ 2,5 |  |  |  |  |  |  | 6,9/nv |  |
|  | peroneal | ne/ne | ≥ 39 | ne/ne | ≥ 2,5 |  |  |  |  |  |  |  |  |
|  | F-Wave |  |  |  |  | ab/ab (ulnar) | 30 ± 2 |  |  |  |  |  |  |
|  |  |  |  |  |  |  |  |  |  |  |  |  |  |

Table 2S. Electroneurographic values for subjects with acute motor axonal neuropathy (AMAN). R/L: Right/Left; ne: not explored; ab: absent; MNCV: motor Nerve conduction Velocity; SNCV: sensitive nerve conduction velocity; CMAP: compound Motor Action Potential; SAP: sensitive action potential; (normal values referred to normative values of our laboratory).
